# Supplementary figures and images for: Temporal Investigation of the Maternal Origins of Fetal Gut Microbiota
Source: Microorganisms. 2024 Sep 9;12(9):1865. doi: 10.3390/microorganisms12091865 (PMC11434507; doi:10.3390/microorganisms12091865)

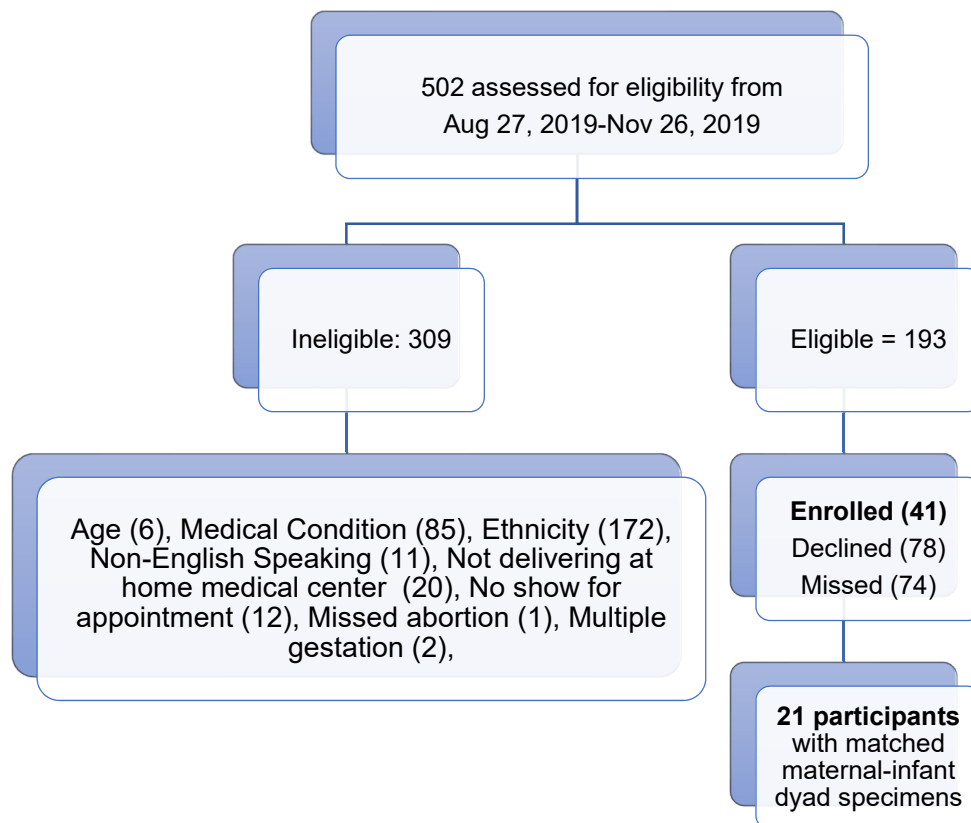

Figure S1. CONSORT diagram of participant assessment for eligibility and enrollment.

Supplement: Supplementary file 1 [file microorganisms-12-01865-s001.zip › microorganisms-3151442-supplementary.pdf]
